# The opposite roles of injustice and cruelty in the internalization of a devaluation: The humiliation paradox revisited

**DOI:** 10.1111/bjso.12823

**Published:** 2024-11-11

**Authors:** Jose A. Gonzalez‐Puerto, Saulo Fernández

**Affiliations:** ^1^ Universidad Nacional de Educación a Distancia (UNED) Madrid Spain

**Keywords:** agency, aggression, cruelty, humiliation, injustice, powerlessness

## Abstract

Cruelty and its link to injustice in contexts of humiliation have not received to date due attention from experimental psychosocial research. Aiming at filling this gap, this paper presents three studies with increasing degrees of experimental control (*N*_total_ = 1098) that show a dual opponent‐process response to being targeted by potentially humiliating actions: while targets appraising more *injustice* internalize less the devaluation underlying the humiliation experience (thus partially dissolving the so‐called “paradox of humiliation”, Fernández et al., 2015, *Personality and Social Psychology Bulletin*, 41, 976), targets appraising more *cruelty* internalize more a devalued self‐view and feel more humiliated. The fine balance between these two closely connected but distinct appraisals is key to understand the internal/subjective experience of targets: seeing themselves mainly as victims of injustice or cruelty will prevent or favour, respectively, their internalization of the devaluation and their feeling humiliated. This opposite pattern also impacts victims' reaction: Both appraisals relate to aggressive responses via anger but while appraising cruelty also paradoxically leads to powerless inertia, appraising injustice (including importantly the injustice of cruelty itself) leads to less powerlessness and more assertive agency. The theoretical and applied implications of approaching the victims of humiliation as victims of both an injustice and a cruelty are discussed.

## INTRODUCTION

Most recent psychosocial research (Elison & Harter, 2007; Elshout et al., 2017; Fernández et al., 2015, 2018, 2022, 2023; Gilbert, 2019; Leask, 2013; Leidner et al., 2012; McCarley, 2009) addresses humiliation as the emotion that accompanies an act of *devaluation* of the target (being, or perceiving oneself as being degraded, devalued, demeaned, lowered), which the victim assesses as *unjust* (undeserved, unfair, lacking legitimacy, justification or proportionality): an ‘illegitimate devaluation’, according to Hardecker (2020). According to this widely held two‐appraisal conceptualization of the emotion of humiliation, victims of humiliating events tend to feel humiliated when they internalize a devaluation of themselves (the *internalization* appraisal) that they consider unjust (the *injustice* appraisal). As victims appraise injustice, anger is also usually present, and as they also internalize a devalued view of themselves, shame is typically reported as well.

Related to this parsimonious view on humiliation, we claim that in many situations another key antecedent may have been overlooked to fully understand the complex (cognitive, emotional and behavioural) experience of humiliation, namely *being, or perceiving oneself as being, a target of cruelty*, that is, the perception that the devaluation is not only unfair but also inflicted with the hostile and malicious intent to cause pain and suffering to the target (Elison & Harter, 2007; Hardecker, 2020). Whenever this cruelty appraisal is salient, we posit it favours targets to feel humiliated. This does not mean that every humiliation is necessarily rooted in cruelty, yet we contend that one of the appalling effects of cruelty (or more precisely, perceiving oneself as a target of a cruel unhuman treatment) is that it facilitates feelings of humiliation in the victim. In Shklar's words (Shklar, 1984, p. 37): ‘What is moral cruelty? It is not just a matter of hurting someone's feelings. It is deliberate and persistent humiliation, so that the victim can eventually trust neither himself nor anyone else’. Despite being often a key component of the humiliating experience, the cruelty appraisal has received much less direct attention than the injustice appraisal. This present work posits that these two different though connected ways of appraising many humiliating situations as unjust and cruel can be empirically disentangled thus showing their distinct—even opposite—effects on the cognitive, emotional and behavioural experience of victims.

By cruelty psychosocial research refers to evil^1^ acts seen as intentionally seeking to produce unjustified harm, a special kind of aggression stemming from the perpetrator/s willingness to destroy and make the victim/s suffer (Baumeister, 2000; Burris, 2022; Phillips, 2011; Piazza et al., 2014; Quiles et al., 2010; Staub, 1989). Building on this conceptualization of cruelty, we propose that when targets perceive that the perpetrators are cruel to them, that is, that the perpetrators deliberately intend to inflict them pain and suffering,^2^ chances increase for them to feel humiliated. This approach is also indebted to Gilbert's contention that while in shame the focus is on the self and the internalization of devalued self‐views, ‘in humiliation the focus is on (what is seen as) unjustifiable devaluation harm and ridicule that's been done by another’ (Gilbert, 2019, p. 421).

### Internalizing the devaluation and externalizing anger in humiliating situations

When the targets appraise a negative treatment against their identity as *just,* the most likely outcome is self‐blame (which favours internalization and shame), whereas if the negative treatment is assessed as *unjust*, the most common outcome is blaming others, which instead prevents internalization and favours anger (Major et al., 2002). Therefore, based on these prior results, the injustice appraisal is expected to protect the victims from internalizing the devaluation when they assess it as unjustified, undeserved and illegitimate. This conclusion parallels a core finding of procedural justice about the higher willingness to accept decisions and rules, even if unpopular, when dictated by an authority whose procedures are perceived to be fair (Tyler, 2006).

Then why do victims of humiliation internalize the devaluation if they assess it as unjust? Why do they not simply externalize it instead through anger? This is the so‐called “paradox of humiliation” (Fernández et al., 2015; see also Jogdand et al., 2020, for a comprehensive review of previous attempts to resolve the paradox). In line with Elison & Harter (2007) (see also Fernández et al., 2018, Study 2), who found that the perceived hostile intent of others do have an effect on how badly we feel about ourselves, we contend that victims' perception of cruel intentions against them is key to understand why they internalize a degraded view of themselves despite assessing their devaluation as unjust.

Cruelty is often facilitated by the perpetrator's dehumanized perception of the victim (Harris & Fiske, 2011). On the targets' side, being treated as less than human, devoid of dignity, unworthy of respect, thus excluded from the moral realm (Opotow, 1990; Staub, 1989), damages their human self‐categorization (Turner et al., 1987), which may then fail to provide its protection against uncertainty at this superordinate level of abstraction (Hogg, 2007). Research on dehumanization (Bastian & Haslam, 2011), objectification (Loughnan et al., 2017) and meta‐dehumanization (Demoulin et al., 2021) provides evidence of the internalization of a devalued self‐view for not being treated as a fellow human. The internalization of this devalued self‐view has been described in terms of reduced self‐esteem (Walker & Knauer, 2011), doubts about who they are, and a worse and more negative view of themselves (Staub, 1989), which guided our operationalization of the internalization appraisal.^3^

Therefore, we hypothesize that while perceptions of cruelty may facilitate the internalization of the devalued self‐view underlying the emotion of humiliation, perceptions of injustice hinder such internalization, thus protecting them from feeling humiliated and ashamed and favouring their anger instead. However, as cruelty itself can be perceived as unjust (for no evil can be fair), the more the injustice component of cruelty is emphasized in the overall appraisal of the victim, the more anger and the less internalization, shame and humiliation will be experienced: the enhancing effect of cruelty on internalization and feelings of humiliation and shame will be counterbalanced by the buffering effect from the perceived injustice of that very same cruelty. Consequently, cruelty typically relates simultaneously to feelings of shame and anger, and so, of humiliation. This mixed emotional state, which is characteristic of many humiliation victims, is thus appropriately called ‘humiliated fury’ (Thomaes et al., 2011). Importantly, this new approach partially dissolves—at the cognitive level of appraisals and particularly in contexts of cruelty—Fernández et al. (2015)'s paradox of humiliation, as injustice does oppose, not favour, the internalization, yet typical targets' reactions to humiliation remain paradoxical.

### Two opposite behavioural responses to humiliation

We additionally contend that by differentiating the injustice from the cruelty component in the victim's appraisal of potentially humiliating situations, and by disentangling their effects in the victim's emotional response, we can also gain a better understanding of the victim's response to those situations. Sluzki (2005) (as cited in Hartling & Lindner, 2016, p. 386) described two styles to respond to humiliating situations: either victims *externalize* their experience, ‘viewing the experience as a personally degrading or insulting event that requires retaliation or revenge’ or *internalize* it ‘as shame, blaming themselves for their experience in a way that prompts hiding behaviour’, thus reacting with inertia (Ginges & Atran, 2008), that is, not engaging in antisocial (e.g. violence) nor prosocial behaviour (e.g. reconciliation), dominated by a feeling of powerlessness (Leidner et al., 2012).

These two possible response styles also reflect, we posit, the opposite effects that we hypothesize from the cruelty and injustice appraisals on internalization: while cruelty would lead more strongly to powerless passive reactions via a devalued identity and humiliation and shame feelings, injustice would oppose powerlessness through its negative effect on the internalization of the devaluation.

None of these two possible outcomes is desirable: neither powerless humiliated victims nor violence fired by aggressive anger. The devastating consequences of this apparent psychological dead end can be sadly traced along the vicious cycle of humiliation and cruelty, powerlessness and violence, characteristic of many intractable conflicts (Lindner, 2006). In the present research, we broaden this view by proposing that, in situations in which we are the target of cruelty, unfairness perceptions and anger may also play a more positive and adaptive role in line with their protective effect highlighted in this paper. Specifically, we hypothesize that judging the humiliating situation as unfair may help the targets not only to minimize their feeling humiliated, but also to stand up for a fair treatment (an *agentic‐assertive* response), as a viable alternative to aggressing back. This hypothesis is in line with recent research on collective action (Renger et al., 2020; Tausch et al., 2011), where the injustice perception was positively related to normative non‐violent collective action and negatively to non‐normative violent collective action. Similarly, we posit that injustice will show a significant positive connection to an agentic‐assertive outcome, a type of reaction that has been found to protect victims of potentially humiliating events from feeling humiliated (Fernández et al., 2022), thus showing that a way out is possible from the apparent psychological dead end of cruelty and humiliation.

In sum, we hypothesize that in potentially humiliating contexts, while the appraisal of injustice (including the injustice of cruelty) prevents the targets from internalizing the devaluation, making them feel more anger but not humiliation nor shame, the appraisal of cruelty facilitates their internalization of a devalued self‐view and makes them feel more humiliated fury (i.e. all the three emotions, humiliation, shame and anger, altogether).

### Overview of the present work

As one feels humiliated when devalued ‘for what one *is* rather than what one *does*’ (Klein, 1991, p. 117), we aimed at probing our propositions along the wide spectrum of possible *identity* threats. Given that our focus is on individual humiliation (i.e. as experienced by individuals), we tested our hypotheses in three different ‘potentially’ humiliating and cruel contexts, each threatening a different aspect of targets' identity (Brewer & Gardner, 1996; Cheek & Cheek, 2018). In Study 1 (correlational), we used a realistic intergroup devaluation of participants' *collective self*, very representative of nowadays extreme political polarization, where participants were made to believe that a well‐known leader of their opposite political orientation had made offensive criticisms on TV against participants' political ideology. In experimental Study 2, we manipulated high versus low potential for humiliation in an imagined interpersonal situation where an acquaintance spreads an unfounded negative gossip against the participants, thus threatening their *relational/public self*. Finally, in experimental Study 3, we manipulated both the cruelty and injustice appraisals and got more conclusive evidence supporting their predicted opposite effects on the subjective/internal experience of humiliation by threatening participants' *individual/personal self* in an imagined interpersonal scenario involving torture. We additionally tested in Study 3 our predictions about the behavioural responses to humiliation.

## STUDY 1

With the aim to test the empirical separability of the cruelty and injustice appraisals and their hypothesized differential roles in the targets' psychological experience of humiliation, we planned a first correlational study in which we devaluated participants' political ideological ingroup to place them in a potentially humiliating situation. As the political ideology is a key component of our collective identities, in a polarized political context like the Spanish one, offensive devaluations of our own positions coming from an important member of the opposite ideology could be perceived as an intentional attack to demean us as an outgroup. Then, we measured the participants' perceptions of cruelty and injustice, together with their levels of internalization of a devalued self‐view and their feelings of anger, shame and humiliation.

### Method

In the three studies included in the present work, we report how we determined our sample size as well as all the data exclusion, manipulations and measures. Data, analysis codes and research materials for the three studies are available from https://osf.io/bkyq3/?view_only=8074a5efb1e047b0a13d214d6b60d870. The data were analysed using SPSS, version 27, and R, version 4.2.2. No data were collected after the data analysis began. The design and analyses of the studies were not preregistered. The National University of Distance Education (UNED) Bioethics Committee approved this research and its method. Participants provided their informed consent to participate voluntarily and anonymously.

#### Participants

We assumed moderate conditions (communalities ranging from .40 to .70 and at least three strong loadings per factor), which, according to Fabrigar and Wegener (2012), would require a minimum sample of 200 participants to obtain reliable factors in an exploratory factor analysis. A total of 655 people over the age of 18 accepted to participate in this online survey. They were recruited by undergraduate students of psychology at the UNED in Spain among their relatives and friends as part of a practical course assignment; the students themselves could not participate in the study. As 59 participants failed to answer correctly to the reading check question, we analysed the responses of *N* = 596 people (58.6% women, *M*_age_ = 35.58, *SD*_age_ = 14.23). In terms of political orientation, 67.6% positioned themselves within the leftist half of the political spectrum, while the rest defined themselves as having a right‐wing political orientation.

#### Procedure

We explained to participants that the study was about the ‘psychological roots of political tension’. Participants completed first a sociodemographic data questionnaire, which included a question about their political orientation where they were asked to position themselves on a 6‐point Likert scale ranging from *very leftist* to *very rightist*, with no central option. Those participants who chose any of the three ‘leftist’ categories (*very*, *moderately* and *slightly*) were considered *leftists* and read the derogatory words spoken by a right‐wing leader against leftists in a prime‐time television programme on a national broadcast channel; otherwise, they were considered *rightists* and read the same offences but spoken by a left‐wing leader.^4^ Participants then answered a questionnaire with the following dependent measures.

#### Measures

All items used in the three studies were 7‐point Likert‐type ranging from 0 (*strongly disagree* or *not at all*) to 6 (*strongly agree* or *extremely*).

##### Emotions

We first asked the participants to respond to a single item for each emotion: ‘To what extent have the words of the intellectual in the TV programme made you feel each of the following emotions?’ The list of emotions included the emotions of interest in this research (i.e. humiliation, anger and shame) plus guilt, embarrassment, humility and pride, which were added to conceal the real research objective.

##### Appraisals

To measure perceptions of *cruelty* (first five items) and *injustice* (last three items), we asked the participants, in random order: ‘To what extent do you think leftists (vs. rightists) [i.e., participants categorized as leftist read “leftists”, whereas those categorized as rightist read “rightists”] have perceived that the words of this well‐known intellectual… (1) have been an act of evil?; (2) have been cruel?; (3) were intended to cause leftists (vs. rightists) harm?; (4) had the intention to hurt leftists (vs. rightists)?; (5) were intended to make leftists (vs. rightists) suffer?; (6) have been unjust?; (7) have been disproportionate?; (8) have been unjustified?’

To measure the level of internalization of a devalued self‐view, we adapted items from previous work in this same research area as those of Fernández et al. (2015, 2018, 2022, 2023). Participants indicated their degree of agreement or disagreement with the following four statements, presented in random order: ‘When reading those words of the intellectual: (9) I saw the idea that leftists (vs. rightists) have of themselves threatened; (10) I had doubts about who leftists (vs. rightists) are; (11) I saw leftists (vs. rightists) in a negative way; (12) I saw reduced leftists' (vs. rightists') collective self‐esteem’.

We run the same preliminary factorial analyses in all the three Studies (see Data S1). As predicted, the model with two separate factors for cruelty and injustice yielded the best fit to the data, thus supporting the empirical separability of these appraisals. Reliabilities for the cruelty, injustice and internalization measures were Cronbach's α = .86, .80 and .82, respectively.

##### Reading check

Participants were asked: ‘Please tell us whether the intellectual who participated in the TV programme was a leftist or a rightist person’. Data from those participants not answering the opposite to their self‐reported political ideology were not analysed. We also asked them to write the intellectual's name in case they had watched that TV programme or been told about it or suspected about who could be. As approximately 30% answered, we presumed most participants believed the offence had really happened.

### Results

Means, standard deviations and intercorrelations are reported in Table 1. Partial correlations showed (in the three studies) that, as expected, both injustice and cruelty relate to internalization, but with opposite signs.

**TABLE 1:** Descriptive statistics and Pearson correlations between appraisals (injustice, cruelty and internalization), emotions (humiliation, anger and shame) and covariates. Study 1.

	*M*	*SD*	Correlations								
			1	2	3	4	5	6	7	8	9
1. Injustice	4.58	1.15	1		−.22***	*.05*	.17***	*.03*	*−.07†*	.11*	*−.04*
2. Cruelty	4.43	1.16	.56***	1	.15***	.17***	.19***	.14***	*.05*	*−.04*	*.05*
3. Internalization	1.60	1.32	−.17***	.03	1						
4. Humiliation	1.57	1.86	.17***	.23***	.24**	1					
5. Anger	2.62	1.92	.32***	.33***	.03	.45***	1				
6. Shame	2.19	2.12	.13***	.19***	.08*	.42***	.38***	1			
7. Political orientation	—	—	.05	−.01	−.01	−.05	−.10*	−.11**	1		
8. Age	35.58	14.23	.10*	.02	−.12**	.06	.03	−.01	.16***	1	
9. Gender	—	—	−.02	.03	.05	.07†	.12**	.07†	−.13**	−.02	1

*Note*: *N* = 596. Political orientation, age and gender used as covariates in the analysis. Gender (1 = male, 2 = female); Political orientation (From −3 = Very leftist to 3 = Very rightist). Data in italics in the first two rows above the diagonal show following partial correlations: First row: Partial correlations between injustice and rest of variables, controlling by cruelty. Second row: Partial correlations between cruelty and rest of variables, controlling by injustice.

†*p* < .10; *p < .05; **p < .01; ***p < .001 (two‐tailed).

**FIGURE 1:**
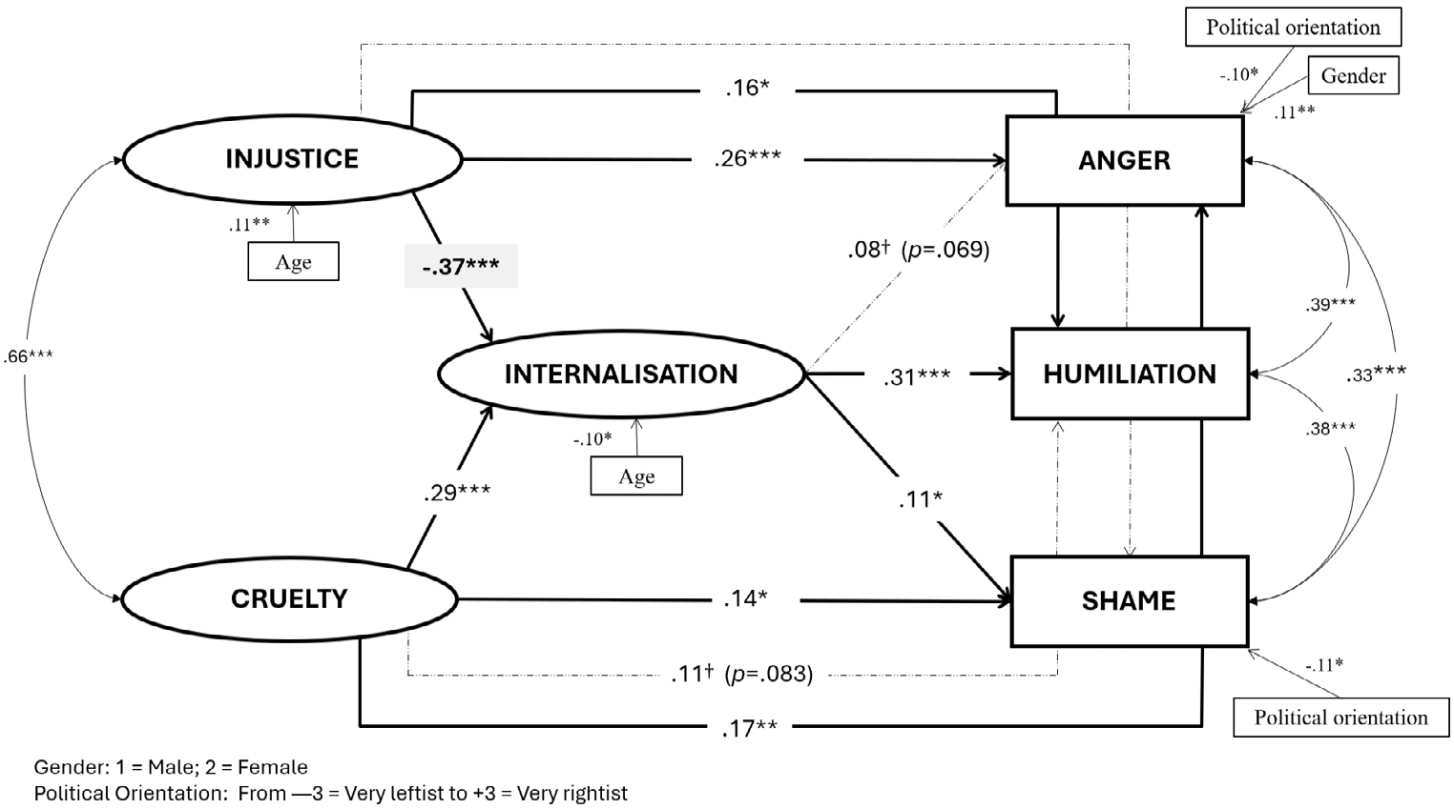
Structural equation model for Study 1. Coefficients are standardized and only provided for significant and marginally significant paths and covariances. Dashed lines indicate non‐significant and marginally significant paths. Only significant covariates are shown. †*p* < .05; *p < .05; **p < .01; ***p < .001.

To test our hypotheses regarding the relationships between appraisals and emotions, we fitted the structural model depicted in Figure 1. Variables in the same vertical were allowed to covariate (injustice and cruelty, on the one hand, and the three emotions, on the other hand). This model yielded an acceptable fit to the observed data: χ^2^ = 297.75, *df* = 105, *p* < .001, χ^2^/*df* = 2.84, CFI = .95, TLI = .92, RMSEA = .055, SRMR = .046.

Because past research had indicated that age and gender may influence these emotions (Hartling & Luchetta, 1999; Secouler, 1992), we included them as full partial covariates in all the three studies. We additionally controlled in this Study 1 by the participant's political orientation, which resulted a significant predictor for both anger and shame (leftists reported higher levels).

Figure 1 highlights the significant paths in the model and Table 2 summarizes the indirect and total effects between the variables, tested—also in Studies 2 and 3—with the Monte Carlo method (Preacher & Selig, 2012), 10^8^ repetitions, using the R package semTools (Jorgensen et al., 2022). Both cruelty and injustice had a significant direct effect on internalization, but as hypothesized, with opposite signs, positive and negative, respectively. These opposite effects on internalization sustained the significant opposite indirect effects (IEs) from cruelty and injustice on humiliation and shame as well; no IE was significant on anger. However, in terms of total effects, only cruelty, not injustice, predicted humiliation and shame feelings; both appraisals, as hypothesized, predicted anger.

**TABLE 2:** Indirect effects (via internalization) and total effects from injustice and cruelty on emotions, Study 1.

	Humiliation	Anger	Shame
IE's via internalization from			
Injustice	**−0.215 [−0.331, −0.116]**	−0.058 [−0.135, 0.005]	**−0.082 [−0.170, −0.008]**
Cruelty	**0.171 [0.081, 0.274]**	0.046 [−0.004, 0.110]	**0.065 [0.006, 0.140]**
Total effects from			
Injustice	0.077 [−0.161, 0.314]	**0.429 [0.195, 0.664]**	0.074 [−0.196, 0.351]
Cruelty	**0.377 [0.147, 0.608]**	**0.367 [0.136, 0.595]**	**0.356 [0.091, 0.623]**

*Note*: Table displays unstandardized coefficients and their 95% confidence intervals, based on 1E8 Monte Carlo repetitions. Significant effects in boldface.

Abbreviation: IE, indirect effects.

### Discussion

Our results indicated that, although perceptions of cruelty and injustice were two closely related constructs, they can and should be differentiated when studying their role in humiliation. These results confirmed that when cruelty and injustice were separated, they related to the internalization of a devalued self‐view in oppositive ways: cruelty facilitated it while injustice prevented it. Also in line with our hypotheses, both appraisals linked to anger, but only cruelty linked to feelings of humiliation and shame.

However, we acknowledge two main limitations in this initial Study: first, we measured participants' *personal* emotions but *collective* appraisals as members of their ingroup. In the next two studies, all measures were done at the personal/individual level. Second, given the correlational nature of our data, no causal relationships could be established. To overcome this limitation, we planned an experimental second study.

## STUDY 2

With the aim of providing additional support for the hurtful (vs. protective) roles of cruelty (vs. injustice) in increasing (vs. preventing) the targets' internalization and emotion of humiliation, we manipulated in an inter‐subject factorial design a negative gossip situation so that it could have high versus low potential to humiliate the participants and measured internalization and emotions (humiliation, anger and shame).

### Method

#### Participants

We used G*Power (Faul et al., 2009) to perform an a priori power analysis for an ANOVA with two groups and 1 degree of freedom (significance level .05, power 80%). We assumed a small to medium effect size (*f* = .17), in line with those reported for internalization in previous works using similar experimental settings, resulting in a minimum sample size of *N* = 274, which also met the minimal sample size requirement of 200 participants to obtain reliable factors in EFA under moderate conditions (Fabrigar & Wegener, 2012). Data collection was stopped when a total of 274 volunteers (60.6% women, *M*_age_ = 35.16 years, *SD*_age_ = 13.84) accepted the invitation to participate in the online survey done by undergraduate students at the UNED in Spain.

#### Procedure

To manipulate the humiliating potential of the situation, we employed two negative gossip scenarios borrowed from Hauke and Abele (2020). Gossip scenarios are a particular effective tool for our research goals, as they constitute a common source of harm in interpersonal interactions (Bandura, 2016), often perceived as hostile acts by the victims that, furthermore, imply publicity, which is a factor that potentiates the feelings of humiliation (Fernández et al., 2023). Hauke and Abele (2020) found that negative gossip is most able to cause harm to the gossip target, as evidenced by the high levels of reputation threat, when it questions the target's morality rather than the target's assertiveness. Based on their findings, we reasoned that the former would be perceived as more potentially humiliating than the latter.

Participants were randomly assigned to a morality gossip condition (*N* = 138) versus an assertiveness gossip condition (*N* = 136), so they had to imagine themselves either as targets of an unfounded negative gossip about their morality or their assertiveness, respectively. Then, participants answered the questionnaire containing the dependent measures.

#### Measures

To measure internalization and the emotions of humiliation, anger and shame, we used the same items as in Study 1, though adapted to the experimental context in Study 2. We also reused Study 1's items 1 to 4 to measure cruelty perceptions, and for injustice we asked (all reverse coded): ‘To what extent do you think… (1) it has been just?; (2) you were as described in the rumour?; (3) you deserved the rumour?’

As in Study 1, factorial analyses confirmed the expected 3‐factor structure for the appraisals, whose reliabilities were Cronbach's α = .87, .74 and .87, for cruelty, injustice and internalization, respectively.

#### Manipulation check

To verify whether our manipulation worked as expected, participants were asked to what extent they found the situation as humiliating. Higher scores in this item indicated that participants found the situation as potentially more humiliating.

### Results

Table 3 shows the zero‐order correlations between all the dependent measures. To assess the effect of the manipulated IV on all DVs, we conducted one‐way ANOVAs on each DV,^5^ whose results are shown in Table 4 together with the descriptive statistics of all the DVs in both conditions. All effects were positive and significant, except for internalization, that was only marginally significant (though it becomes significant when controlling by age and gender) and showed an unexpected negative effect (i.e. less internalization in the higher potentially humiliating condition). As indicated by their difference in effect size, this manipulation seems to have produced a bigger impact on the appraisal of injustice (leading to lower internalization) than on the appraisal of cruelty.

**TABLE 3:** Descriptive statistics and Pearson correlations between appraisals (injustice, cruelty and internalization), emotions (humiliation, anger and shame) and covariates. Study 2.

	*M*	*SD*	Correlations							
			1	2	3	4	5	6	7	8
1. Injustice	5.39	0.79	1		−.36***	−.14*	.16**	−.22***	*−.00*	*.05*
2. Cruelty	3.95	1.30	.32***	1	.20***	.28***	.34***	.25***	*−.05*	*.12†*
3. Internalization	1.51	1.47	−.32***	.08	1					
4. Humiliation	2.30	1.84	−.05	.25***	.46***	1				
5. Anger	3.61	1.64	.27***	.40***	.15*	.48***	1			
6. Shame	1.89	1.75	−.14*	.19***	.50***	.70***	.30***	1		
7. Age	35.16	13.84	−.02	−.06	−.24***	−.23***	−.18**	−.21***	1	
8. Gender	—	—	.09	.14*	.08	.15*	.24***	.07	−.06	1

*Note*: *N* = 274 (except for correlations with Gender, *N* = 273). Gender (1 = male, 2 = female). Data in italics in the first two rows above the diagonal show following partial correlations: First row: Partial correlations between injustice and rest of variables, controlling by cruelty. Second row: Partial correlations between cruelty and rest of variables, controlling by injustice.

†*p* < .10; *p < .05; **p < .01; ***p < .001 (two‐tailed).

**TABLE 4:** Descriptive statistics and results of IV one‐way ANOVAs on each dependent variable, Study 2.

	Descriptive statistics		ANOVAs *F*(1, 272)		
	Mean (SD)		Main effects		
	Humiliating potential				
	Low	High	*F*	*p*	$\eta_{p}^{2}$
Injustice	5.13 (0.89)	5.64 (0.58)	**31.58**	**<.001**	**.104**
Cruelty	3.73 (1.34)	4.17 (1.23)	**7.74**	**.006**	**.028**
Internalization	1.69 (1.53)	1.35 (1.40)	3.68	.056	.013
Humiliation	1.93 (1.60)	2.66 (1.98)	**11.10**	**<.001**	**.039**
Anger	3.09 (1.66)	4.12 (1.45)	**29.95**	**<.001**	**.099**
Shame	1.56 (1.51)	2.22 (1.91)	**10.20**	**.002**	**.036**
Manipulation check	3.01 (1.85)	3.79 (1.74)	**12.74**	**<.001**	**.045**

*Note*: Significant effects in boldface.

#### Structural equation model

**FIGURE 2:**
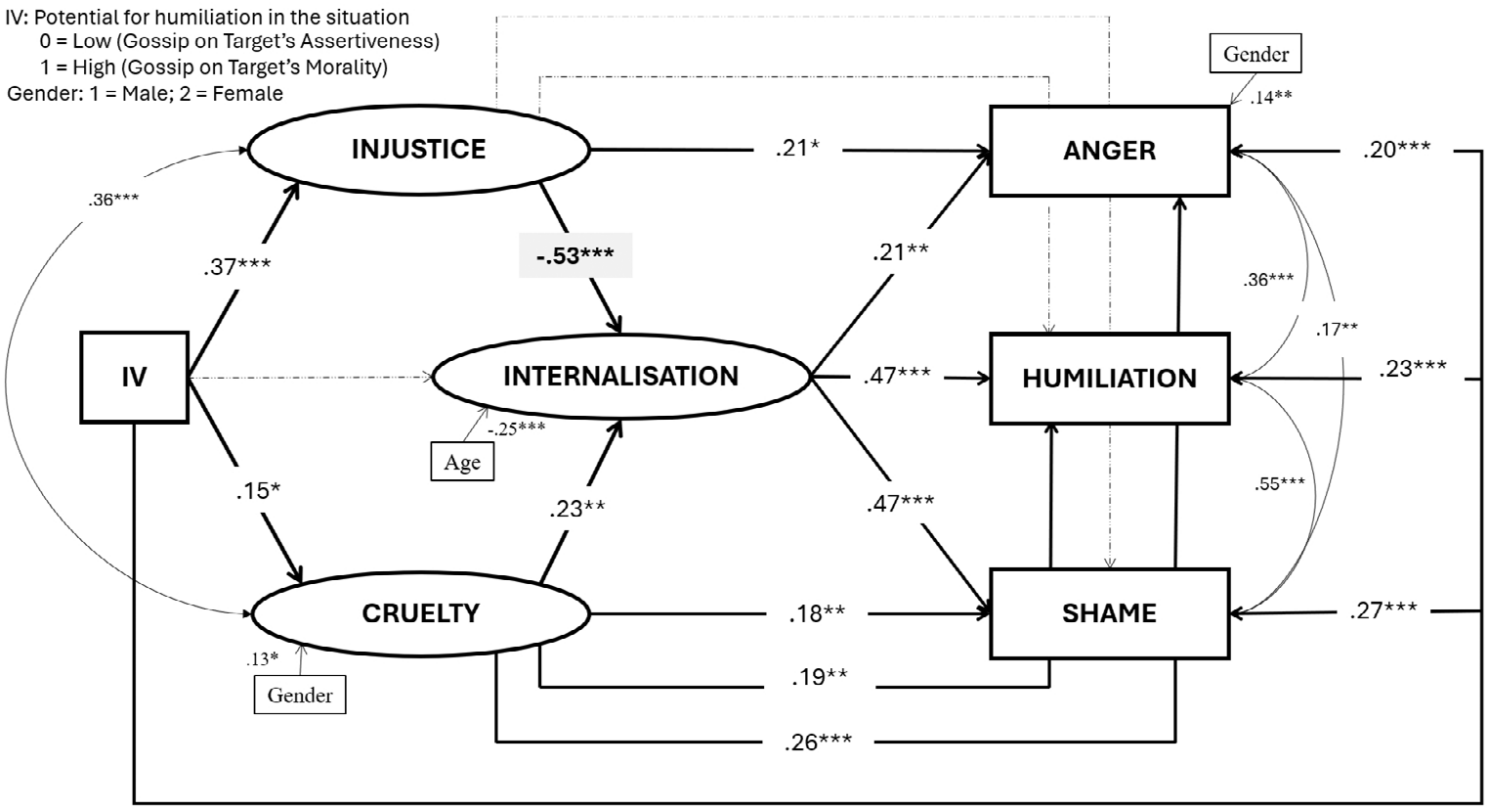
Structural equation model for Study 2. Coefficients are standardized and only provided for significant and marginally significant paths and covariances. Dashed lines indicate non‐significant and marginally significant paths. Only significant covariates are shown. †*p* < .05; *p < .05; **p < .01; ***p < .001.

As in Study 1, we fitted the structural model depicted in Figure 2 to test our hypotheses about the relationships between appraisals and emotions, yielding an acceptable fit to the observed data (χ^2^ = 183.81, *df* = 89, *p* < .001, χ^2^/*df* = 2.07, CFI = .95, TLI = .93, RMSEA = .062, SRMR = .057). Table 5 shows the results of testing the indirect effects (IEs) in this fitted model. As expected, the manipulated independent variable had significant and opposite IEs on internalization via the appraisals of injustice and cruelty, negative and positive, respectively. These two opposite IEs also span through internalization to emotions, positively via cruelty and negatively via injustice. Additionally, while the cruelty appraisal on its own also mediated the positive effect of the IV on all the three emotions, the injustice appraisal on its own just mediated the positive effect of the IV on anger.

**TABLE 5:** Indirect effects from the IV on internalization and emotions (humiliation, anger and shame) via the appraisals (injustice, cruelty and internalization). Study 2.

Indirect effects from IV via	Internalization	Humiliation	Anger	Shame
Injustice	**−0.464 [−0.726, −0.246]**	−0.018 [−0.256, 0.217]	**0.254 [0.047, 0.511]**	−0.170 [−0.419, 0.047]
Injustice–Internalization		**−0.333 [−0.543, −0.167]**	**−0.134 [−0.271, −0.039]**	**−0.315 [−0.509, −0.158]**
Cruelty	**0.082 [0.007, 0.184]**	**0.099 [0.008, 0.232]**	**0.123 [0.014, 0.265]**	**0.089 [0.005, 0.211]**
Cruelty–Internalization		**0.059 [0.005, 0.136]**	**0.024 [0.001, 0.062]**	**0.055 [0.005, 0.128]**

*Note*: Table displays unstandardized coefficients and their 95% confidence intervals, based on 1E8 Monte Carlo repetitions. Significant effects in boldface. *N* = 273.

### Discussion

Study 2 replicated the main findings of Study 1. Cruelty and injustice appraisals are related to each other but can and should be distinguished to unveil their differential roles in the complex experience of humiliation: cruelty facilitating humiliation and shame feelings, and injustice playing a protective role by preventing or minimizing the internalization of the devaluation, and so also reducing the humiliation and shame feelings. Contrary to our predictions, the effect of the IV (high vs. low potentially humiliating situation) on internalization was only marginally significant and negative, which we argue could be due to a manipulation that was appraised more as unjust than as cruel, in consistency with anger and humiliation mean values being, respectively, above and below the central points of their scales. Nevertheless, contrary to our theorizing, this lower internalization did not translate into lower levels of humiliation, what we think could be related, as indicated by the significant direct effect of IV on humiliation, to different psychological mechanisms not included in this parsimonious model of humiliation.

Interestingly, our results showed how the opposite effects of cruelty (positive/harming) and injustice (negative/protective) on internalization can cancel each other out, and even being possible for the injustice perceptions to prevail. This result has important practical implications for the clinical and psychosocial interventions with (potential) victims of humiliations: by making them aware of the unjust/undeserved aspects of the aggression, we may help them to deal with the internalization driven by the mere fact of having been cruelly treated by a fellow human. We will return to this issue in the General Discussion.

Our manipulation of the gossip type served as an indirect and joint manipulation of both the cruelty and the injustice appraisals. We had no control on its separate effects on each of the appraisals and it resulted in a manipulation more effective on eliciting injustice than cruelty perceptions. We solved this limitation in Study 3, where we designed a separate manipulation of cruelty and injustice that helped us to identify their distinct effects in a more conclusive way.

## STUDY 3

The goal of this third study was to experimentally test our hypotheses in a laboratory study, in which we aimed to isolate the effect of cruelty from that of injustice. To that end, we designed a 2 Injustice (Unjust vs. Just) × 2 Cruelty (Cruel vs. Non‐cruel) inter‐subject factorial design in which we manipulated independently the level of injustice and cruelty of a potentially humiliating scenario (an arrest by the police) and measured the levels of internalization and emotions. Additionally, we measured the victim's behavioural responses to test our hypotheses regarding the victim's response contingent on the manipulated independent variables and the cognitive‐emotional process.

### Method

#### Participants

We used G*Power (Faul et al., 2009) to perform an a priori power analysis for an ANOVA with four groups and one degree of freedom (significance level .05, power to 80%). We assumed a small to medium effect size (*f* = .20, as in previous works), giving us a minimum sample size of *N* = 199. To account for the exclusion of incomplete participations and those failing to pass the Reading Check, we aimed at 15% additional participations. A total of 230 undergraduate psychology students at the UNED in Spain participated in the study; all of them passed the Reading Check^6^ but two did not complete the questionnaire, so we finally analysed the data of 228 volunteers (80.7% women, *M*_age_ = 32.20, *SD*_age_ = 11.24).

#### Procedure

Once participants provided their informed consent and completed a small questionnaire about their sociodemographic characteristics, we asked them to read a text that described a situation that they had to imagine as if it was really happening to them. Specifically, they were randomly assigned to imagine a situation where they were arrested, either being innocent (unjust condition) versus guilty (just condition) of the crime they were accused of. Likewise, they were also randomly assigned to imagine that the treatment they received from the police officers was either ‘violent and offensive, sometimes bordering on torture’ (cruel condition), versus ‘correct and polite, sometimes bordering on cordiality’ (non‐cruel condition). Participants then answered the questionnaire with the measures described below.

#### Measures

As in the previous studies, we measured the key appraisals (i.e. injustice, cruelty and the level of internalization) and discrete emotions (humiliation, anger and shame). Because in the present study we manipulated the level of injustice and cruelty in a quite direct way, we used the appraisals of injustice and cruelty as manipulation checks. We used the same items as in the previous studies to measure the discrete emotions, internalization and cruelty (5 items, as in Study 1). To measure injustice, and in order to improve its reliability (acceptable but low in Study 2), we focused on the deservingness and lack of justification sides of the measure and asked the participants, in random order: ‘To what extent do you agree or disagree with the following statements regarding your arrest and the officers' conduct?: (1) It is unjust; (2) No person deserves to experience such a situation; (3) I deserve it –reverse coded–; (4) It is justified–reverse coded–’.

As in the previous two studies, factorial analyses (see Data S1) confirmed the 2‐factor structure for cruelty and injustice. Reliabilities were Cronbach's α = .83, .96 and .87 for the injustice, cruelty and internalization measures, respectively.

We further measured three types of target's responses.^7^ To that end, participants responded to what extent they saw themselves in this situation reacting in each of the following ways—presented in random order—: [*Aggression;* α = .80] (1) with physical aggression, (2) insulting, (3) taking revenge; [*agency‐assertiveness;* α = .88] (4) claiming for a fair treatment; (5) demanding my rights; (6) defending myself; (7) protesting when I am not respected; and [*powerlessness;* α = .85] (8) unable to respond; (9) blocked; (10) with no power whatsoever to act; (11) having no will to respond.

### Results

Table 6 shows the zero‐order correlations between all the dependent measures.^8^

**TABLE 6:** Descriptive statistics and Pearson correlations between the appraisals (injustice, cruelty and internalization), emotions (humiliation, anger and shame), behavioural responses (aggression, assertive agency and powerlessness) and covariates (age and gender). Study 3.

	*M*	*SD*	Correlations										
			1	2	3	4	5	6	7	8	9	10	11
1. Injustice (M.C.)	4.18	1.75	1		−.37***	*.03*	.29***	−.18**	.24***	*.08*	*−.11*	−.14*	*.07*
2. Cruelty (M.C.)	3.36	2.21	.61***	1	.35***	.34***	.20**	*.11*	*.02*	.28***	.34***	*.00*	*−.05*
3. Internalization	3.10	1.78	−.20**	.15*	1								
4. Humiliation	4.50	1.81	.28***	.43***	.38***	1							
5. Anger	4.66	1.45	.47***	.43***	−.00	.44***	1						
6. Shame	3.96	1.99	−.15*	−.01	.57***	.45***	.23*	1					
7. Assertive agency	4.38	1.46	.31***	.20**	−.32***	.04	.30***	−.29***	1				
8. Aggression	1.36	1.43	.29***	.39***	−.03	.22***	.35***	−.07	.22***	1			
9. Powerlessness	3.62	1.66	.13†	.34***	.62***	.56***	.23***	.51***	−.21***	.01	1		
10. Age	32.20	11.24	−.17*	−.10	−.13†	−.14*	−.10	−.11†	.04	−.05	−.15*	1	
11. Gender	—	—	.05	−.01	.13†	.09	.06	.16*	−.03	−.15*	.13†	−.18**	1

*Note*: *N* = 228. Gender (1 = male, 2 = female). Data in italics in the first two rows above the diagonal show following partial correlations: First row: Partial correlations between injustice and rest of variables, controlling by cruelty. Second row: Partial correlations between cruelty and rest of variables, controlling by injustice.

†*p* < .10; *p < .05; **p < .01; ***p < .001 (two‐tailed).

To assess the effect of the manipulated IVs on all DVs, we first conducted two‐way Injustice × Cruelty ANOVAs on each DV. Their results and the descriptive statistics of all the DVs in all four conditions are shown in Table 7 and described in the following two sections.

**TABLE 7:** Descriptive statistics and results of the 2 Cruelty (Cruel vs. Non‐cruel situation) × 2 Injustice (Unjust vs. Just situation) ANOVAs on each dependent variable (manipulation checks: cruelty and injustice appraisals; internalization; emotions: humiliation, anger and shame; and behavioural responses: aggression, assertive agency and powerlessness), Study 3.

	Descriptive statistics: Mean (SD)				Cruelty × injustice ANOVAs. *F*s(1224)								
					Main effects						Interaction		
	Unjust		Just		Cruelty			Injustice					
	Cruel	Non‐cruel	Cruel	Non‐cruel	*F*	*p*	$\eta_{p}^{2}$	*F*	*p*	$\eta_{p}^{2}$	*F*	*p*	$\eta_{p}^{2}$
Injustice (M.C.)	5.26 (1.01)	4.60 (1.39)	4.50 (1.31)	2.17 (1.57)	**71.54**	**<.001**	**.242**	**82.43**	**<.001**	**.269**	**22.62**	**<.001**	**.092**
Cruelty (M.C.)	5.03 (1.35)	1.76 (1.53)	4.92 (1.07)	1.01 (1.09)	**454.87**	**<.001**	**.670**	**6.48**	**.012**	**.028**	3.77	.053	.017
Internalization	2.74 (1.73)	1.83 (1.51)	3.99 (1.58)	3.62 (1.50)	**9.13**	**.003**	**.039**	**51.22**	**<.001**	**.186**	1.60	.207	.007
Humiliation	5.10 (1.55)	3.90 (2.08)	5.00 (1.43)	3.78 (1.86)	**27.94**	**<.001**	**.111**	0.22	.642	.001	0.00	.960	.000
Anger	5.21 (1.23)	4.75 (1.56)	4.84 (1.13)	3.72 (1.52)	**19.05**	**<.001**	**.078**	**14.89**	**<.001**	**.062**	3.37	.068	.015
Shame	3.37 (2.32)	3.44 (2.04)	4.43 (1.70)	4.57 (1.51)	0.18	.672	.001	**18.35**	**<.001**	**.076**	0.02	.887	.000
Assertive Agency	4.79 (1.40)	4.62 (1.18)	4.36 (1.44)	3.72 (1.56)	**4.64**	**.032**	**.020**	**12.62**	**<.001**	**.053**	1.56	.213	.007
Aggression	1.88 (1.70)	0.92 (1.20)	1.78 (1.33)	0.65 (0.86)	**34.89**	**<.001**	**.135**	1.05	.308	.005	0.21	.649	.001
Powerlessness	3.73 (1.71)	2.79 (1.77)	4.45 (1.30)	3.26 (1.43)	**26.19**	**<.001**	**.105**	**8.23**	**0.005**	**.035**	0.35	.554	.002

*Note*: Significant effects in boldface.

#### Manipulation check

ANOVAs on the injustice appraisal yielded significant positive main effects (with comparable effect sizes) of injustice and cruelty manipulations, and a significant interaction. ANOVAs on the cruelty appraisal yielded significant positive main effects of the injustice and cruelty manipulations (the former with a much lower effect size), and a non‐significant interaction effect. As shown in Table 7, all cruel conditions, no matter if just or unjust, are perceived as highly unjust, what means that in the cruel‐just condition the high levels of injustice come from cruelty itself, as no evil can be fair. However, the unjust‐non‐cruel condition displays high levels of injustice but low levels of cruelty (injustice may not be cruel), thus depicting an asymmetrical pattern between these two appraisals. These results confirmed that our manipulation had worked within the expected outcomes.

#### Main effects on internalization, emotions and Behavioural responses

ANOVAs on internalization yielded significant main effects of injustice and cruelty (negative and positive, respectively), and a non‐significant interaction, thus confirming our main hypothesis regarding the opposite effect both appraisals exert on internalization.

Regarding the discrete emotions, as expected, we found a significant positive main effect of the cruelty manipulation (but not of the injustice manipulation) on humiliation, with no significant interaction. In line with our theorizing, results on anger showed positive main effects of injustice and cruelty, and a non‐significant interaction. Regarding shame, we found a significant negative main effect of the injustice manipulation, with no significant effect from the cruelty manipulation nor a significant interaction.

Regarding the behavioural responses, we found significant positive main effects of cruelty on all the three responses, significant main effects of injustice on agency‐assertiveness and powerlessness (positive and negative, respectively) but not on aggression, and non‐significant interaction effects on all three measures.

#### Indirect effects on internalization, emotions and Behavioural responses

**FIGURE 3:**
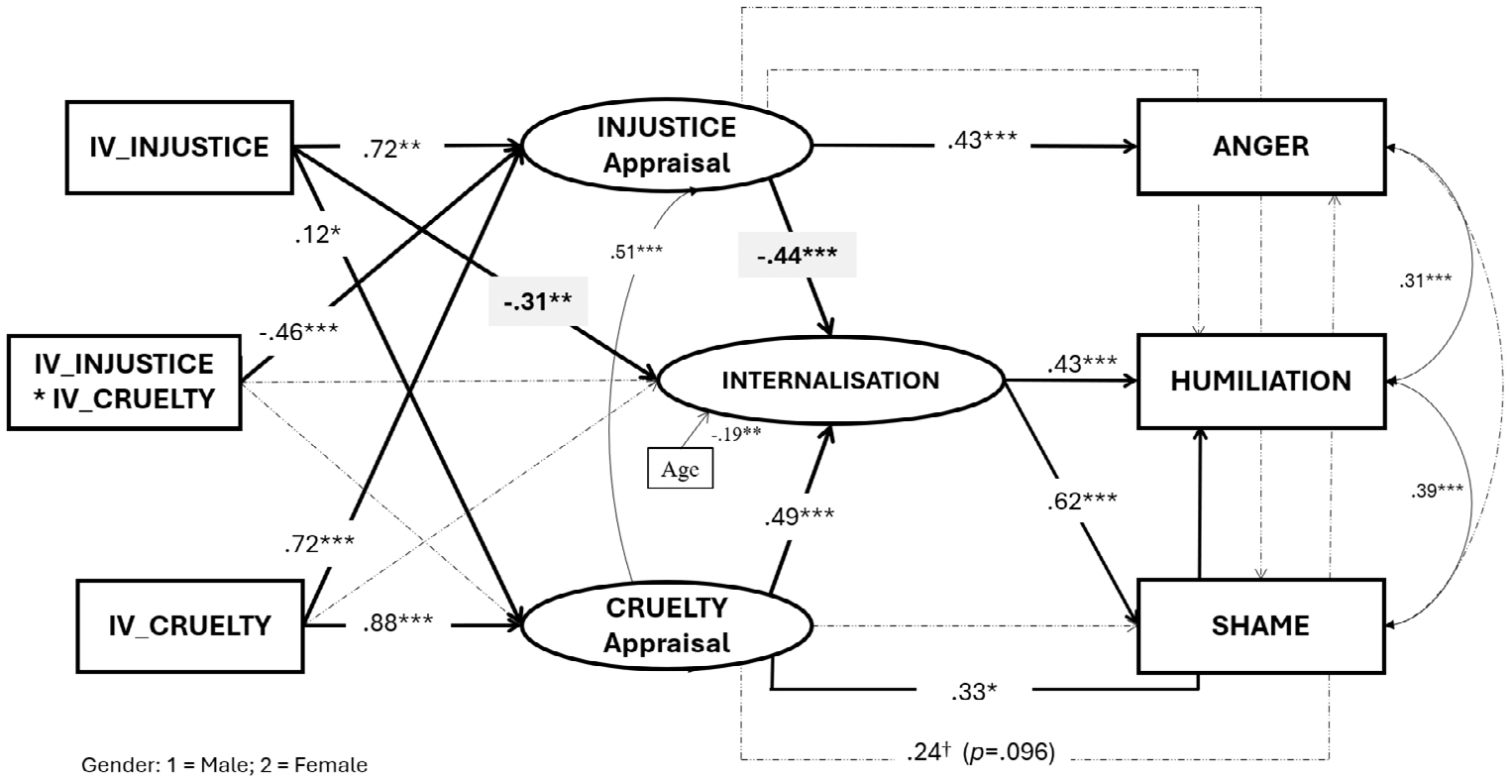
Structural equation model for Study 3. Coefficients are standardized and only provided for significant and marginally significant paths and covariances. Dashed lines indicate non‐significant and marginally significant paths. Only significant covariates are shown. †*p* < .05; *p < .05; **p < .01; ***p < .001. As none of the nine paths between IVs (including the interaction term) to any emotion was significant or marginally significant, they were not included in the Figure.

To test our hypotheses regarding the relationships between the manipulated factors and appraisals and emotions, we finally fitted the model depicted in Figure 3 (injustice and cruelty were allowed to covariate among them). The model showed an acceptable fit to the observed data: χ^2^ = 294.47, *df* = 142, *p* < .001, χ^2^/*df* = 2.07, CFI = .95, TLI = .93, RMSEA = .069, SRMR = .058.

As summarized in Table 8, and nicely replicating our results in the previous two studies, we found that both manipulations presented the same pattern regarding their IEs on internalization: a negative IE (i.e. opposing internalization) when mediated by the injustice appraisal, and a positive IE (i.e. favouring internalization) when mediated by the cruelty appraisal. As predicted, these opposite IEs also reached the emotions: whereas both manipulations favoured humiliation and shame when mediated by cruelty‐internalization, both manipulations opposed humiliation and shame when mediated by injustice‐internalization. No IEs were found significant via internalization on anger. Additionally, both manipulations had a significant positive IE on anger via the appraisal of injustice, but only the manipulation of cruelty presented a significant positive IE on humiliation via the appraisal of cruelty.

**TABLE 8:** Indirect effects from the IVs (Injustice and Cruelty) on Internalization and Emotions (Humiliation, Anger and Shame) via the appraisals (Injustice, Cruelty and Internalization). Study 3.

	Internalization	Humiliation	Anger	Shame
IEs from IV Injustice via appraisals				
Injustice	**−0.789 [−1.299, −0.332]**	0.483 [−0.134, 1.131]	**0.896 [0.368, 1.470]**	0.045 [−0.620, 0.700]
Injustice–Internalization		**−0.496 [−0.888, −0.192]**	−0.092 [−0.281, 0.047]	**−0.778 [−1.327, −0.326]**
Cruelty	**0.142 [0.005, 0.334]**	0.139 [−0.005, 0.334]	0.082 [−0.019, 0.242]	0.020 [−0.121, 0.178]
Cruelty–Internalization		**0.090 [0.003, 0.216]**	0.017 [−0.009, 0.062]	**0.141 [0.005, 0.332]**
IEs from IV_Cruelty via Appraisals				
Injustice	**−0.787 [−1.293, −0.335]**	0.483 [−0.135, 1.120]	**0.894 [0.371, 1.454]**	0.045 [−0.614, 0.694]
Injustice–Internalization		**−0.495 [−0.880–0.194]**	−0.091 [−0.281, 0.047]	**−0.777 [−1.310, −0.327]**
Cruelty	**1.066 [0.421, 1.723]**	**1.040 [0.152, 1.927]**	0.613 [−0.107, 1.345]	0.151 [−0.793, 1.085]
Cruelty–Internalization		**0.671 [0.247, 1.169]**	0.124 [−0.067, 0.374]	**1.053 [0.414, 1.752]**

*Note*: Table displays unstandardized coefficients and their 95% confidence intervals, based on 1E8 Monte Carlo repetitions. Significant effects in boldface.

Indirect effects on behavioural responses are described in detail in Data S1. Supporting our predictions, while IEs via anger from both manipulations (injustice and cruelty) resulted significant and positive on aggression, only the whole IE from the injustice IV was significant (and positive) on agency. Also as expected, a clear pattern of opposite IEs emerged on powerlessness: negative from the injustice manipulation (via internalization and shame, thus the injustice appraisal), positive from the cruelty manipulation (via the cruelty appraisal and humiliation).

### Discussion

The separate manipulation of cruelty and injustice in Study 3 provided overall empirical support for our hypotheses, replicating main results from the previous two studies. As expected, cruel situations were also appraised as largely unjust, while unjust situations were appraised as cruel only to a much lesser extent. Our experimental manipulation of cruelty and injustice showed that these two appraisals can and should be differentiated: though related to each other, their roles are quite different, even opposite. Cruelty appears as a key element for the experience of humiliation, closely connected to their prototypical behavioural responses in the victims (aggression and powerlessness). On the other hand, the injustice appraisal reveals a more positive and ‘well‐adjusted’ role than was previously attributed: Injustice perceptions help the victims to avoid internalizing the devaluation, thus escaping from powerlessness, and instead having the possibility to respond resorting to agentic‐assertive responses.

## GENERAL DISCUSSION

This work posited and found in all its three studies that injustice and cruelty, though closely related constructs, can be empirically distinguished so that their differential effects on the experience of humiliation are disentangled: while cruelty positively predicted the internalization of the devaluation being inflicted upon the targets, thus playing a crucial role leading to their feelings of humiliation and shame, the injustice perceived in the humiliating situation helped them instead to minimize internalization and feel anger but not humiliation nor shame. In Study 3, we additionally explored their effects on the behavioural responses and found that while the injustice appraisal led to agentic‐assertive responses, the cruelty appraisal did not; and while cruelty also positively predicted powerlessness, injustice did not (in fact, it was negatively related to powerlessness). Moreover, cruelty—but not injustice—was related to aggressive responses. All in all, we have shown that, particularly in situations that imply cruelty, injustice is not by itself the key antecedent of humiliation that it has often thought to be, whereas cruelty does play a key role in triggering humiliation. In fact, in cruel scenarios, perceptions of injustice (importantly, including the injustice of cruelty itself) may protect the victims, minimizing their internalization of the devaluation and their feelings of humiliation and shame. We have also found in Study 3 the well‐established connection between anger and aggression, but as predicted, we have shown a more well‐adaptive side of anger, which can lead not only to aggressive responses, but also to healthier agentic‐assertive ones to restore our own self‐views.

These results shed new light on the paradox of humiliation (Fernández et al., 2015) in cruelty contexts: Assessing the devaluation as unjust hinders, not favours, its internalization, and mainly leads to anger but not to humiliation. Furthermore, this work posited and found that appraising cruelty does favour such internalization and feelings of humiliation. Therefore, when cruelty is taken into account, a significant part of the original paradox simply dissolves. Nevertheless, we still require further understanding of the paradoxical behavioural responses associated simultaneously to cruelty in humiliating situations: powerless inertia and aggression (Leidner et al., 2012).

Although our results have shown that cruelty does not always ‘merely increase hatred and make people less willing to surrender or capitulate’ (Baumeister, 2000, p. 116), but can also lead to the internalization of a devalued self‐view and powerlessness, further understanding of the psychological mechanisms underlying this process is still necessary. As we theorized in the introduction, one possibility worth exploring is whether these negative effects could be related to a process of self‐dehumanization. The experimental study of the link between humiliation and dehumanization is an interesting domain to be explored further in novel lines of research.

Our results may seem to contradict previous work on attributions of historical wrongs to evil essence, as they go with more hostility to the perpetrators and not more internalization (Imhoff et al., 2017). In the same vein, Miller et al. (2002) have argued that dispositional attributions for evil are more consistent with condemning the perpetrator. According to this interpretation, victims seek for an attribution of bad treatment, and knowing that the perpetrator is a habitually evil person would deflect the attribution from their own behaviours. We think both pieces of evidence can be reconciled: perceptions of cruelty do facilitate the internalization of the devaluation but at the same time, this effect can be compensated or even exceeded by other effects, like the overall perception of injustice, notably including the injustice associated to the perceived cruelty, especially when adding the perspective of time and the processing of memories about the original humiliation. Our research could also be considered as additional evidence of the paradoxical role of cruelty, leading both to anger and humiliation/shame, powerless inertia and aggression.

We acknowledge that the imagined scenarios used in studies 2 and 3 cannot fully account for the cognitive, emotional and behavioural responses of any individual facing a real scenario. Although we chose this specific experimental manipulation for ethical reasons, and we succeeded in finding consistent support for our theorizing, future research should attempt to replicate these results with alternative experimental settings available to research on humiliation (memory recall, cyberball, etc.) and with real victims of humiliation/cruelty in more ecological contexts. Another possible limitation of this research may be the use of single‐item measures for the emotions (humiliation, anger and shame). We acknowledge further research is needed to confirm their validity and reliability, though these emotions are so familiar and their meaning so distinctive that we think they will likely be included in the category of ‘constructs that are unidimensional, clearly defined, and narrow in scope’, and therefore, potentially as valid and reliable as their multi‐item counterparts (Allen et al., 2022).

Our hypotheses regarding the individual experience of humiliation found support both in interpersonal (Studies 2 and 3) and intergroup contexts (Study 1). Future research could investigate whether this model also extends to collective humiliation (i.e. as experienced by groups/collectives), and structural humiliation. In the former, mutual assessing of cruelty and injustice may lead to the perpetuation of cycles of humiliation and violence. Particularly in strong asymmetric intergroup conflicts, we posit that the more vulnerable group may suffer the humiliation that follows the cruelty they appraise both in the dominant group and, more broadly, in the international community who witnesses the situation without supporting them. In the latter, the lack of a clear perpetrator to whom to attribute evil intentions may result in the unfairness appraisal playing the central role as antecedent.

From an applied perspective, our dual opponent process towards the internalization of a devalued self‐view contributes to the literature reflecting on healing ‘the hurt of humiliation’ (Hartling & Lindner, 2016; Hartling & Luchetta, 1999; Klein, 1991; Trumbull, 2008), a long‐lasting psychological harm on the victims' identity, especially detrimental to their dignity and self‐respect (Margalit, 1996; Shklar, 1984; Statman, 2000). Clinical and psychosocial interventions with (potential) victims of humiliation, both individuals and collectives, may benefit from emphasizing the injustice appraisal. Though cruelty devastation will likely require specific work addressing post‐traumatic symptoms, our work suggests that training victims in asserting unfairness/injustice judgements with regard to the treatment being received, whereby their attention is distracted from being targets of evil/cruelty, may help them to resort to more well‐adaptive agentic‐assertive responses to stand up for their basic rights, in consistency with the protective role that has recently been described for agency on victims of humiliation (Fernández et al., 2022). These responses, though, are mainly driven by anger, which can also lead to aggression, thus requiring proper emotional regulation to avoid re‐entering the cruelty–humiliation cycle.

This work showed the potential of cruelty to humiliate its victims but also the power of asserting the injustice of cruelty to counterbalance its appalling effects. Evil carries its own weakness within, for no evil can be fair. Victims can always learn to respect themselves through protesting the injustice upon them, not necessarily through violence, but hopefully via healthier agentic‐assertive strategies.

## AUTHOR CONTRIBUTIONS

**Jose A. Gonzalez‐Puerto:** Conceptualization; methodology; software; data curation; supervision; formal analysis; validation; investigation; funding acquisition; writing – original draft; visualization; writing – review and editing; project administration; resources. **Saulo Fernández:** Conceptualization; methodology; funding acquisition; supervision; validation; visualization; writing – review and editing.

## FUNDING INFORMATION

This research and the preparation of this material were supported by the Universities Ministry's Grant FPU19/01189 to Jose A. Gonzalez‐Puerto and the project PID2022‐138915NB‐I00 (Principal Researcher: Saulo Fernández) from the Ministry of Science and Innovation.

## CONFLICT OF INTEREST STATEMENT

The authors declared no potential conflicts of interest with respect to the research, authorship, and/or publication of this article.

## Supporting information


Data S1.

## Data Availability

The data that support the findings of this study are openly available in OSF at https://osf.io/bkyq3/?view_only=8074a5efb1e047b0a13d214d6b60d870.

## Footnotes

^1^
Adjective “evil” applied to people also means “that they are very wicked by nature and take pleasure in doing things that harm other people” (https://www.collinsdictionary.com/dictionary/english/evil).

^2^
Definition of cruelty in the online Collins dictionary (https://www.collinsdictionary.com/dictionary/english/cruelty).

^3^
The role of internalization in humiliation has recently been challenged by Jogdand et al. (2020). However, their conceptualisation of internalization seems to differ from that followed here as it is unlikely that their counterexamples (i.e. people not feeling humiliated despite internalizing the devaluation) would score any high in the measure for internalization operationalized as indicated.

^4^
The texts of the press clippings and the vignettes used in the three studies can be found in Data S1.

^5^
ANCOVAS on each DV, with age and gender as covariates, were also run with no change on these results. Same applies to Study 3. See Data S1.

^6^
Reading Check details in Data S1.

^7^
Factorial analysis available in Data S1.

^8^
A more detailed description of Study 3 results in Data S1.
