# Supplementary material for: The opposite roles of injustice and cruelty in the internalization of a devaluation: The humiliation paradox revisited
Source: Br J Soc Psychol. 2024 Nov 11;64(2):e12823. doi: 10.1111/bjso.12823 (PMC11927379; doi:10.1111/bjso.12823)
Supplement: Supplementary file 1 — Data S1. [file BJSO-64-0-s001.docx]

************

**SUPPORTING INFORMATION**

********

**ADDITIONAL ANALYSES FOR STUDY 1**

**Study 1: Exploratory Factor Analysis (Appraisals & Emotions)**

In order to test our measurement model, we split our sample into two random subsets of *N_1_* = 200 and *N_2_* = 396 participants, so we could run separate exploratory and confirmatory factor analyses (EFA, CFA), respectively. A principal components EFA with oblimin rotation was run with *N_1_* on the 15 items (5 for cruelty, 3 for injustice, 4 for internalisation, and 1 item for each of the three emotions -humiliation, anger and shame-). It detected the expected four factors (eigenvalues > 1), explaining 67.28% of the variance. Factor loadings ranged from .49 to .91 (cruelty), .71 to .88 (injustice), .78 to .82 (internalisation), and .65 to .79 (negative emotions: humiliation, anger, and shame), cross-loading absolute values not above .35, .11, .16, and .19, respectively. Reliabilities for the cruelty, injustice, and internalisation measures in sample *N_1_* were Cronbach’s α = .87, .80, and .82, respectively (Cronbach’s α = .86, .80, and .82 for the whole sample *N* = 596). Correlations between each pair of emotions ranged between .34 and .39 in sample *N_1_*, *p*’s < .001. The detection of the two factors for cruelty and injustice provided initial support for our hypothesis on the empirical separability of these appraisals.

**Study 1: Confirmatory Factor Analysis (Appraisals & Emotions)**

We further tested the factorial structure of this measurement model in sample *N_2_* with CFA using the R package lavaan (version 0.6–12, Rosseel, 2012). We compared the four-factor scheme above with all possible alternative 1, 2 or 3 factor-solutions that could be formed by accordingly putting together the items of the 4 factors, or of 2, or 3 factors. Only the four-factor model showed an acceptable fit (i.e., both CFI and TLI above .90) to the observed data (χ^2^ = 226.46, *df* = 84, χ^2^/*df* = 2.70, *p* < .001, CFI = .94, TLI = .92, RMSEA = .065, SRMR = .063); all other models, including those with injustice and cruelty as a single latent construct, showed significant poorer fits in their respective chi-squared difference tests (*p*s < .001). More interestingly, the model we theorized with 6 factors (i.e., humiliation, anger, and shame as 3 different factors) yielded a significant better fit (χ^2^ = 192.98, *df* = 78, χ^2^/*df* = 2.47, *p* < .001, CFI = .95, TLI = .93, RMSEA = .061, SRMR = .053) than the initial 4-factor model (chi-squared difference test, *p* < .001).

**ADDITIONAL ANALYSES FOR STUDY 2**

**Study 2: Exploratory Factor Analysis (Appraisals & Emotions)**

A principal component EFA with direct oblimin rotation run on the 14 items (4 for cruelty, 3 for injustice, 4 for internalisation, and 1 item for each of the three emotions -humiliation, anger and shame-), yielded the expected four factors (eigenvalues > 1) for cruelty, injustice, internalisation, and the set of three emotions (humiliation, anger, and shame), explaining 72.02% of the variance. Factor loadings ranged from .63 to .94 (cruelty), .73 to .83 (injustice), .81 to .87 (internalisation), and .73 to .88 (emotions: humiliation, anger, and shame), with cross-loading absolute values not higher than .22, .11, .16, and .22, respectively.

**Study 2: Confirmatory Factor Analysis (Appraisals & Emotions)**

As in Study 1, we further ran a CFA on this four-factor measurement model (14 items). Results showed an acceptable fit to the observed data: χ^2^ = 213.59, *df* = 71, χ^2^/*df* = 3.01, *p* < .001, CFI = .92, TLI = .90, RMSEA = .086, SRMR = .091. Replicating the results of Study 1, while all alternative 1, 2 or 3 factor-models, including those with cruelty and injustice as a single latent variable, showed significant poorer fits (chi-squared difference tests, *p*s < .001), the 6 factor-model (with humiliation, anger, and shame as 3 different factors) yielded a significant better fit (χ^2^ = 153.80, *df* = 65, χ^2^/*df* = 2.37, *p* < .001, CFI = .95, TLI = .93, RMSEA = .071, SRMR = .062) than the 4-factor model (chi-squared difference test, *p* < .001).

**Study 2: ANCOVAS**

We conducted a series of ANCOVAs with the appraisals (injustice, cruelty, and internalisation), and the three emotions (humiliation, anger, and shame) as the dependent variables, and age and gender as covariates. Results from these analyses are shown in Table 1 at the end of this document.

Both, age and gender, were significant covariates for the emotions (humiliation and anger) and the Manipulation Check. Age was also a significant covariate for internalisation and shame, and gender for cruelty, but none of them was a significant covariate for injustice. However, inclusion of these covariates did not remove any main effect of the IV on the dependent variables that had been significant in the main text ANOVA analyses, and additionally, the marginally significant negative effect of IV on internalisation became significant. As argued in the main text, this unexpected negative effect may be due to the manipulation being more clearly of injustice than of cruelty.

**ADDITIONAL ANALYSES FOR STUDY 3**

**Study 3: Exploratory Factor Analysis (Appraisals & Emotions)**

A principal component EFA with oblimin rotation run on the 16 items (5 for cruelty, 4 for injustice, 4 for internalisation, and 1 item for each of the three emotions -humiliation, anger and shame-), yielded the expected three factors (eigenvalues > 1) for the appraisals (injustice, cruelty, and internalisation).

Humiliation and anger constituted a fourth factor for emotions (eigenvalue = .828), and shame loaded on both internalisation (.58) and the emotions factor (.46). Altogether these 4 factors explained 75.57% of the variance. Factors loadings ranged from .73 to .96 (cruelty), .61 to .91 (injustice), .72 to .91 (internalisation, without shame), and .73 to .75 (just humiliation and anger) with cross-loading absolute values not higher than .26, .30, .19, and .25 respectively.

**Study 3: Confirmatory Factor Analysis (Appraisals & Emotions)**

As in the previous two studies, we further ran a CFA on the measurement model based on these 16 items. Replicating the results of Studies 1 and 2, the 4-factor model (with the three emotions in one single factor) showed a fit to the observed data (χ^2^ = 353.13, *df* = 98, *p* < .001, χ^2^/*df* = 3.60, CFI = .91, TLI = .88, RMSEA = .107, SRMR = .123), not really acceptable but nevertheless significantly better than any alternative one-, two-, or three-factor solution (chi-squared difference tests, *p*s < .001), including all possible models with the cruelty and injustice items together in the same factor.

However, our theorized 6-factor model with the three appraisal factors (injustice, cruelty, and internalisation) plus a separate factor for each emotion (humiliation, anger, and shame) yielded an acceptable fir to the observed data: χ^2^ >= 229.72, *df* = 92, *p* < .001, χ^2^/*df* = 2.50, CFI = .95, TLI = .93, RMSEA = .081, SRMR = .066.

**Study 3: Exploratory Factor Analysis (Behavioural Responses)**

An EFA with oblimin rotation of the 11 items detected the expected three factors (eigenvalues > 1), explaining 72.54% of the variance. Factor loadings ranged from .81 to .91 (aggression), .80 to .91 (agency-assertiveness), and .79 to .85 (powerlessness), with cross-loading absolute values not higher than .15, .15, and .14, respectively. The Cronbach’s alphas of these three measures were .80, .88, and .85 for aggression, agency-assertiveness, and powerlessness, respectively.

**Study 3: ANCOVAS**

We conducted a series of ANCOVAs with the appraisals (injustice and cruelty), internalisation, emotions (humiliation and anger), and behavioural reactions (assertive agency, aggression, and powerlessness) as the dependent variables and age and gender as covariates. Results from these analyses are shown in Table 2.

While age was only a significant covariate for internalisation, gender was only a significant covariate for aggression. Inclusion of these covariates did not remove any main effect of the IVs on the dependent variables that had been significant in the original analyses as reported in the main text.

**Study 3: Manipulation Check (Results).**

**Refer to Table 7 in main text**

Results on the injustice appraisal yielded a significant main effect of injustice, a significant main effect of cruelty, and a significant interaction. Participants appraised higher levels of injustice in the unjust (*M* = 4.98, *SD* = 1.23) than in the just condition (*M* = 3.43, *SD* = 1.85) and in the cruel (*M* = 4.88, *SD* = 1.22) than in the non-cruel condition (*M* = 3.32, *SD* = 1.92). The interaction effect was accounted by a more pronounced effect of the injustice manipulation for participants in the non-cruel condition, *t*(89) = 8.85, *p* < .001, *d* = 1.86, than in the cruel condition, *t*(102.15) = 4.15, *p* < .001, *d* = .78. As seen in Table 7, all cruel conditions, no matter if just or unjust, are perceived as highly unjust, what means that in the cruel-just condition the high levels of injustice come from cruelty itself, as no evil can be fair, However, the unjust-non cruel condition displays high levels of injustice but low levels of cruelty (injustice may not be cruel), thus depicting an asymmetrical pattern between these two appraisals,

Results on the cruelty appraisal yielded a significant main effect of the injustice manipulation, a significant main effect of the cruelty manipulation, and a nonsignificant interaction effect. Participants appraised significantly higher levels of cruelty in the unjust (*M* = 3.61, *SD* = 2.16) and cruel (*M* = 4.97, *SD* = 1.22) conditions than in the just (*M* = 3.11, *SD* = 2.24) and non-cruel conditions (*M* = 1.36, *SD* = 1.36), respectively. These results confirmed that our manipulation had worked within the expected outcomes.

**Study 3: Effects of the IVs (Cruelty and Injustice) on Internalisation, Emotions, and Behavioural Responses (Results)**

**Refer to Table 7 in main text**

Results on internalisation yielded significant main effects of injustice and cruelty, and a nonsignificant interaction. Confirming our main hypothesis regarding the opposite effect that injustice and cruelty exert on internalisation, whereas participants reported significantly *lower* levels of internalisation in the unjust (*M* = 2.34, *SD* = 1.69) as compared to the just condition (*M* = 3.81, *SD* = 1.55), they reported significantly *higher* levels of internalisation in the cruel (*M* = 3.36, *SD* = 1.77) as compared to the non-cruel condition (*M* = 2.77, *SD* = 1.75).

Regarding the discrete emotions, we found a significant main effect of the cruelty manipulation (but not of the injustice manipulation) on humiliation, with no significant interaction. As expected, participants in the cruel condition reported higher levels of humiliation feelings (*M* = 5.05, *SD* = 1.49) than participants in the non-cruel condition (*M* = 3.83, *SD* = 1.96). Results on anger showed main effects of injustice and cruelty, and a nonsignificant interaction. In line with our theorizing, participants in both the unjust and the cruel conditions reported to have experienced higher levels of anger (*M* = 5.01, *SD* = 1.40 and *M* = 5.02, *SD* = 1.19, respectively) than participants in the just and non-cruel conditions (*M* = 4.32, *SD* = 1.43 and *M* = 4.21, *SD* = 1.62, respectively). Regarding shame, we found a significant negative main effect of the injustice manipulation (but not of the cruelty manipulation), with no significant interaction. Consistent with our hypotheses, participants in the unjust condition reported lower levels of shame (*M* = 3.40, *SD* = 2.20) than participants in the just condition (*M* = 4.50, *SD* = 1.61).

Regarding the behavioural responses, we found significant positive main effects of cruelty on all the three responses, significant main effects of injustice on agency-assertiveness and powerlessness (positive and negative, respectively) but not on aggression, and nonsignificant interaction effects on all three measures. Participants in the cruel condition reacted with significantly more aggression (*M* = 1.83, *SD* = 1.52), agency-assertiveness (*M* = 4.57, *SD* = 1.43), and powerlessness (*M* = 4.08, *SD* = 1.56) than participants in the non-cruel condition (*M* = 0.78, *SD* = 1.04; *M* = 4.14, *SD* = 1.46; *M* = 3.04, *SD* = 1.61, respectively). As expected, participants in the unjust condition reacted with more agency (*M* = 4.72, *SD* = 1.30) and less powerlessness (*M* = 3.32, *SD* = 1.79) than participants in the just condition (*M* = 4.06, *SD* = 1.53; *M* = 3.90, *SD* = 1.48, respectively).

**Study 3: Indirect Effects on the Behavioural responses**

**via the Appraisals and Emotions**

To test our hypotheses regarding the relationships between the manipulated factors and behavioural responses via appraisals and emotions, we finally fitted an extension of the previous model that included the responses as outcome variables, with appraisals and emotions as mediators (injustice and cruelty were allowed to covariate among them, and so were the three emotions). The model, depicted in Figure 3 below, showed and acceptable fit to the observed data: χ^2^ = 686.37, *df* = 381, *p* < .001, χ^2^/*df* = 1.80, CFI = .93, TLI = .92, RMSEA = .059, SRMR = .060. Main indirect effects are summarized in Table 3.

Supporting our predictions, indirect effects from both manipulations outlined two quite opposite patterns with regards to behavioural responses: (the sum of all) IEs from both the injustice and cruelty IVs resulted significant and positive on aggression (mainly via anger). Regarding agency, only (the sum of all) IEs from the injustice IV was significant: although both IVs showed significant and positive IEs on agency via the injustice appraisal and anger, only the injustice IV additionally showed a significant positive IE via internalisation, which emphasizes the important role that the appraisal of internalization plays in weighting the other two appraisals and react to their imbalance). Finally, while (the sum of all) IEs from the injustice IV was significant and negative on powerlessness, (the sum of all) IEs from the cruelty IV was not significant in this study: nevertheless, the expected opposite pattern on powerlessness can be seen in Table 3 below, where only negative significant IEs (red ones) appear from the injustice IV (via internalisation and shame), and only positive significant IEs (black ones) appear from the cruelty IV (in this case, via the cruelty appraisal and humiliation).

| **Table 1** | | | | | | | | | | | | | | | | |  | | | | |  | | |  | | |  | | |  | | |  | | |  | |
| --- | --- | --- | --- | --- | --- | --- | --- | --- | --- | --- | --- | --- | --- | --- | --- | --- | --- | --- | --- | --- | --- | --- | --- | --- | --- | --- | --- | --- | --- | --- | --- | --- | --- | --- | --- | --- | --- | --- |
|  | | | | | | | | | | | | | | | | |  | | | | |  | | |  | | |  | | |  | | |  | | |  | |
| ***Descriptive Statistics and Results of IV One-way ANCOVAs on Each Dependent Variable, with Age and Gender as Covariates. Study 2*** | | | | | | | | | | | | | | | | | | | | | | | | | | | | | | | | | | | | | | |
|  | Descriptive Statistics | | |  | | ANCOVAs  *F*(1, 269) | | | | | | | | | | | | | | | | | | | | | | | | | | | | | | | | |
|  | Mean (SD) | | |  | |  | | | |  | | |  | | |  |  | | | | |  | | |  | | |  | | |  | | |  | | |  | |
|  |  | | |  | | IV Main Effects | | | | | | | | | | | COVARIATES | | | | | | | | | | | | | | | | | | | | | |
|  | Humiliating Potential | | |  | |  | | | |  | | |  | | |  | AGE | | | | | | | | | | |  | | | GENDER | | | | | | | |
|  | LOW | HIGH | |  | | *F* | | *p* | | | *η_p_^2^* | | |  | | | | *F* | | *p* | | | *η_p_^2^* | | |  | | | *F* | | | *p* | | | *η_p_^2^* | | |  |
| Injustice | 5.15 (0.88) | | 5.64 (0.58) | |  | | **28.85** | | **< .001** | | | **.097** | | |  | | | | 0.03 | | .873 | | | .000 | | |  | | | 1.05 | | | .306 | | | .004 | | |
| Cruelty | 3.74 (1.34) | | 4.17 (1.23) | |  | | **5.94** | | **.015** | | | **.022** | | |  | | | | 0.39 | | .534 | | | .001 | | |  | | | **4.18** | | | **.042** | | | **.015** | | |
| Internalisation | 1.68 (1.53) | | 1.35 (1.40) | |  | | **6.29** | | **.013** | | | **.023** | | |  | | | | **18.07** | | **< .001** | | | **.063** | | |  | | | 1.87 | | | .172 | | | .007 | | |
| Humiliation | 1.93 (1.61) | | 2.66 (1.98) | |  | | **8.07** | | **.005** | | | **.029** | | |  | | | | **11.39** | | **< .001** | | | **.041** | | |  | | | **4.41** | | | **.037** | | | **.016** | | |
| Anger | 3.09 (1.66) | | 4.12 (1.45) | |  | | **24.71** | | **< .001** | | | **.084** | | |  | | | | **5.43** | | **.021** | | | **.020** | | |  | | | **13.96** | | | **< .001** | | | **.049** | | |
| Shame | 1.55 (1.51) | | 2.22 (1.91) | |  | | **8.06** | | **.005** | | | **.029** | | |  | | | | **9.61** | | **.002** | | | **.034** | | |  | | | 0.52 | | | .473 | | | .002 | | |
| Manipulation Check | 3.00 (1.85) | | 3.79 (1.74) | |  | | **9.18** | | **.003** | | | **.033** | | |  | | | | **9.93** | | **.002** | | | **.036** | | |  | | | **18.09** | | | **< .001** | | | **.063** | | |
| *Note.* Significant effects in boldface. | | | | | | | | | | | | | | | | |  | | | | |  | | |  | | |  | | |  | | |  | | |  | |

*N*=273, as participants with gender = 3 (“Other”; just 1 case) had to be filtered out in order not to distort the results.

| **Table 2** | | | | | | | | | | | | | | | | | | | |  |
| --- | --- | --- | --- | --- | --- | --- | --- | --- | --- | --- | --- | --- | --- | --- | --- | --- | --- | --- | --- | --- |
|  | | | | | | | | | | | | | | | | | |  |  |  |
| ***Descriptive Statistics and Results of the 2 CRUELTY (Cruel vs. Non cruel situation) x 2 INJUSTICE (Unjust vs. Just situation) ANCOVAs on each Dependent Variable (Manipulation Checks: Cruelty & Injustice Appraisals; Internalisation; Emotions: Humiliation, Anger, and Shame; and Behavioural Responses: Aggression, Assertive Agency, and Powerlessness), with Age and Gender as covariates. Study 3*** | | | | | | | | | | | | | | | | | | | |  |
|  | Cruelty x Injustice ANCOVAs. *F*s(1,222) | | | | | | | | | | | | | | | | | | |  |
|  | Main effects | | | | | | |  | Interaction | | |  | COVARIATES | | | | | | |  |
|  | Cruelty | | |  | Injustice | | |  | Cruelty x Injustice | | |  | AGE | | |  | GENDER | | |  |
|  | *F* | *p* | *η_p_^2^* |  | *F* | *p* | *η_p_^2^* |  | *F* | *p* | *η_p_^2^* |  | *F* | *p* | *η_p_^2^* |  | *F* | *p* | *η_p_^2^* |  |
| Injustice (M.C.) | **70.87** | **< .001** | **.242** |  | **82.38** | **< .001** | **.271** |  | **20.92** | **< .001** | **.086** |  | 1.54 | .215 | .007 |  | 2.41 | .122 | .011 |  |
| Cruelty (M.C.) | **448.21** | **< .001** | **.669** |  | **6.70** | **.010** | **.029** |  | 3.46 | .064 | .015 |  | 0.00 | .988 | .000 |  | 0.95 | .331 | .004 |  |
| Internalisation | **8.44** | **.004** | **.037** |  | **53.25** | **< .001** | **.193** |  | 2.34 | .128 | .010 |  | **5.07** | **.025** | **.022** |  | 1.93 | .166 | .009 |  |
| Humiliation | **27.52** | **< .001** | **.110** |  | 0.21 | .644 | .001 |  | 0.02 | .878 | .000 |  | 1.85 | .175 | .008 |  | 2.48 | .117 | .011 |  |
| Anger | **19.00** | **< .001** | **.079** |  | **15.05** | **< .001** | **.063** |  | 2.89 | .090 | .013 |  | 0.30 | .584 | .015 |  | 1.69 | .195 | .008 |  |
| Shame | | 0.25 | .620 | .001 |  | **18.79** | **< .001** | **.078** |  | 0.15 | .696 | .001 |  | 3.27 | .072 | .001 |  | 3.14 | .078 | .014 |
| Assertive Agency | **5.08** | **.025** | **.022** |  | **13.15** | **< .001** | **.056** |  | 1.71 | .192 | .008 |  | 1.27 | .260 | .006 |  | 0.20 | .888 | .000 |  |
| Aggression | **32.68** | **< .001** | **.128** |  | 0.68 | .409 | .003 |  | 0.34 | .562 | .002 |  | 0.23 | .632 | .001 |  | **4.72** | **.031** | **.021** |  |
| Powerlessness | **25.69** | **< .001** | **.104** |  | **8.50** | **.004** | **.037** |  | 0.13 | .724 | .001 |  | 3.30 | .070 | .015 |  | 2.98 | .086 | .013 |  |
| *Note.* Significant effects in boldface. | | | | | | | | | | | | | | | | | |  |  |  |


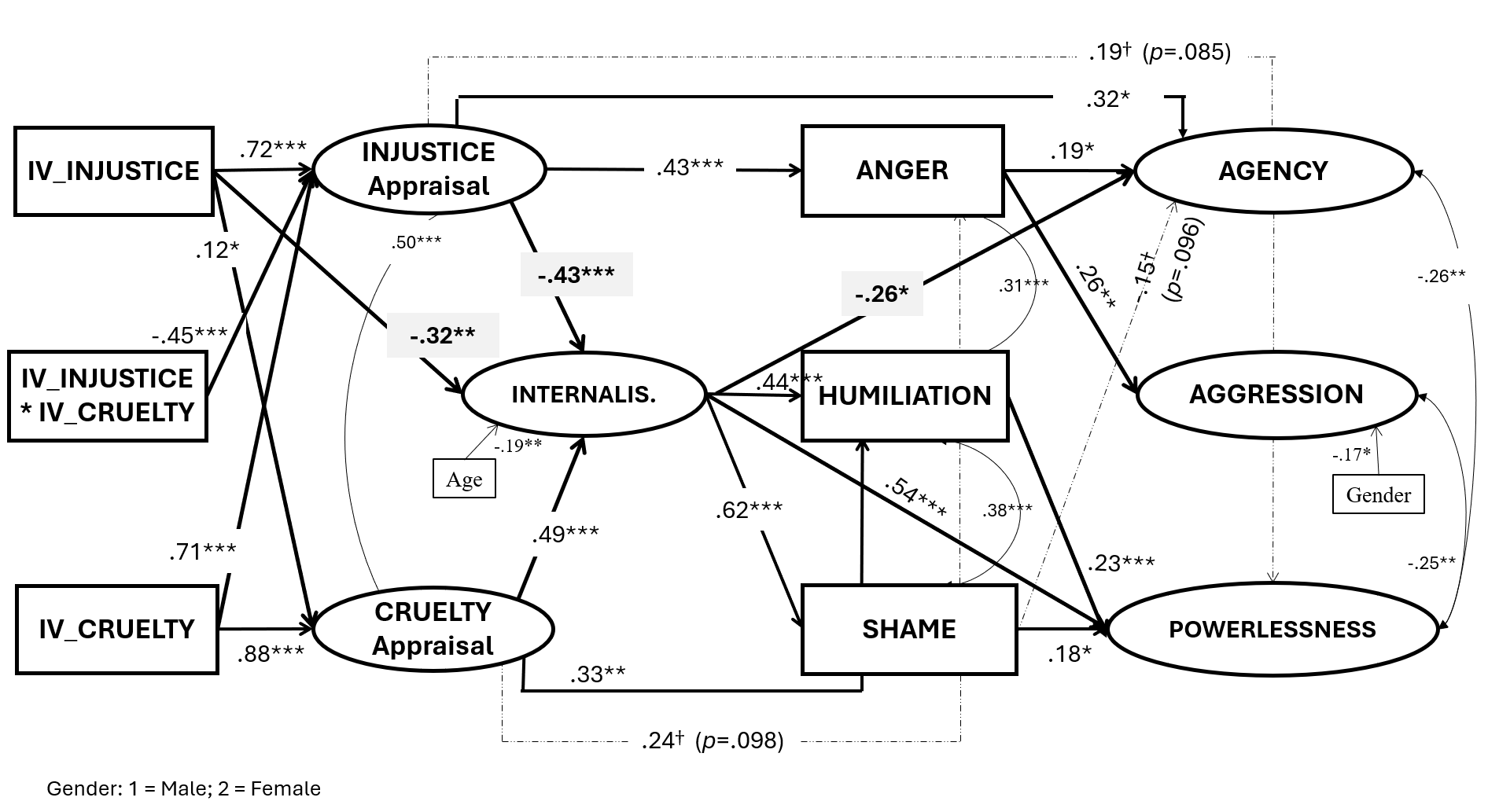


**Figure 1. Structural Equation Model (with Behavioural Responses), Study 3**

Note: Coefficients are standardized and only provided for significant and marginally significant paths and covariances.

Dashed lines indicate marginally significant paths.

Only significant covariates are shown.

† *p* < .05; * *p* < .05; ** *p* < .01; *** *p* < .001

| **Table 3**  ***Indirect Effects (IE’s) from IVs (Cruelty and Injustice) on Behavioural Responses (B.R.) Study 3*** | | | | | |  |
| --- | --- | --- | --- | --- | --- | --- |
|  | | | | | |  |
|  | AGENCY |  | AGGRESSION |  | POWERLESSNESS | |
| **Indirect Effects from IV_INJUSTICE** |  |  |  |  |  | |
| Total IE via Injustice (1+2+3+4+5+29+30+31) | **0.932 [0.440, 1.458]** |  | 0.280[-0.168, 0.748] |  | -0.199 [-0.824, 0.414] | |
| Total IE via Cruelty (6+7+8+9+10+32+33+34) | -0.059 [-0.192, 0.030] |  | 0.065 [-0.026, 0.206] |  | 0.165 [-0.001, 0.413] | |
| Total IE via Internalisation (2+7+21+[29..34]+[41..43]) | **0.413 [0.159, 0.707]** |  | 0.136 [-0.120, 0.407] |  | **-1.469 [-2.122, -0.892]** | |
| Total IE via Humiliation (3+8+22+29+32+41) | 0.003 [-0.048, 0.064] |  | 0.004 [-0.051, 0.067] |  | 0.015 [-0.138, 0.177] | |
| Total IE via Anger (4+9+23+30+33+42) | **0.157 [0.024, 0.336]** |  | **0.219 [0.062, 0.424]** |  | 0.022 [-0.132, 0.180] | |
| Total IE via Shame (5+10+24+31+34+43) | 0.105 [-0.017, 0.272] |  | 0.042 [-0.093, 0.192] |  | **-0.185 [-0.401, -0.030]** | |
| SUM of IEs from IV_INJUSTICE | **1.138 [0.675, 1.636]** |  | **0.477 [0.042, 0.922]** |  | **-0.742 [-1.412, -0.081]** | |
| Direct Effect (DE) from IV_INJUSTICE | -0.311 [-0.885, 0.261] |  | -0.256 [-0.854, 0.346] |  | 0.056 [-0.598, 0.699] | |
| TOTAL Effect (IEs+DE) from IV_INJUSTICE | **0.827 [0.334, 1.317]** |  | 0.221 [-0.266, 0.706] |  | **-0.686 [-1.371, -0.002]** | |
|  |  |  |  |  |  | |
| **Indirect Effects from IV_CRUELTY** |  |  |  |  |  | |
| Total IE via Injustice (11+12+13+14+15+35+36+37) | **0.928 [0.440, 1.445]** |  | 0.279 [-0.169, 0.742] |  | -0.198 [-0.825, 0.418] | |
| Total IE via Cruelty (16+17+18+19+20+38+39+40) | -0.445 [-1.086, 0.211] |  | 0.484 [-0.188, 1.158] |  | **1.239 [0.322, 2.182]** | |
| Total IE via Internalisatn (12+17+25+[35..40]+[44..46]) | -0.051 [-0.198, 0.074] |  | -0.017 [-0.097, 0.036] |  | 0.182 [-0.260, 0.639] | |
| Total IE via Humiliation (13+18+26+35+38+44) | 0.057 [-0.075, 0.207] |  | 0.061 [-0.078, 0.218] |  | **0.253 [0.077, 0.488]** | |
| Total IE via Anger (14+19+27+36+39+45) | **0.170 [0.029, 0.352]** |  | **0.238 [0.076, 0.448]** |  | 0.024 [-0.139, 0.193] | |
| Total IE via Shame (15+20+28+37+40+46) | 0.020 [-0.047, 0.109] |  | 0.008 [-0.039, 0.073] |  | -0.035 [-0.168, 0.077] | |
| SUM of IEs from IV_CRUELTY | 0.482 [-0.066, 1.022] |  | **0.679 [0.125, 1.244]** |  | 0.720 [-0.021, 1.469] | |
| Direct Effect (DE) from IV_CRUELTY | 0.108 [-0.524, 0.742] |  | 0.355 [-0.324, 1.027] |  | 0.480 [-0.255, 1.211] | |
| TOTAL Effect (IEs+DE) from IV_CRUELTY | **0.590 [0.125, 1.041]** |  | **1.034 [0.561, 1.499]** |  | **1.200 [0.558, 1.865]** | |
| Notes in next page. | | | | | |  |

*Note.* Table displays unstandardized coefficients and their 95% confidence intervals, based on 1E8 Monte Carlo repetitions.

Significant effects in boldface: Negative ones in red.

HM = Humiliation; AN = Anger; SH = Shame; B.R. = Behavioural Responses; Appr = Appraisal; IV = Independent Variable

Numbering of IEs:

| 1.IV_Injustice–InjusticeAppr-B.R.  2.IV_Injustice–InjusticeAppr-Internalisation-B.R.  3.IV_Injustice–InjusticeAppr-Humiliation-B.R.  4.IV_Injustice–InjusticeAppr-Anger-B.R.  5.IV_Injustice–InjusticeAppr-Shame-B.R.  6.IV_Injustice–CrueltyAppr-B.R.  7.IV_Injustice–CrueltyAppr-Internalisation-B.R.  8.IV_Injustice–CrueltyAppr-Humiliation-B.R.  9.IV_Injustice–CrueltyAppr-Anger-B.R.  10.IV_Injustice–CrueltyAppr-Shame-B.R.  29.  IV_Injustice–InjusticeAppr-Internalisat-HM-B.R.  30.  IV_Injustice–InjusticeAppr-Internalisat-AN-B.R.  31.  IV_Injustice–InjusticeAppr-Internalisat-SH-B.R.  32.  IV_Injustice–CrueltyAppr-Internalisat-HM-B.R.  33.  IV_Injustice–CrueltyAppr-Internalisat-AN-B.R.  34.  IV_Injustice–CrueltyAppr-Internalisat-SH-B.R. | 11.IV_Cruelty–InjusticeAppr-B.R.  12.IV_Cruelty–InjusticeAppr-Internalisation-B.R  13.IV_Cruelty–InjusticeAppr-Humiliation-B.R.  14.IV_Cruelty–InjusticeAppr-Anger-B.R.  15.IV_Cruelty–InjusticeAppr-Shame-B.R.  16.IV_Cruelty–CrueltyAppr-B.R.  17.IV_Cruelty–CrueltyAppr-Internalisation-B.R.  18.IV_Cruelty–CrueltyAppr-Humiliation-B.R.  19.IV_Cruelty–CrueltyAppr-Anger-B.R.  20.IV_Cruelty–CrueltyAppr-Shame-B.R.  35.  IV_Cruelty –InjusticeAppr-Internalisat-HM-B.R.  36.  IV_Cruelty –InjusticeAppr-Internalisat-AN-B.R.  37.  IV_Cruelty –InjusticeAppr-Internalisat-SH-B.R.  38.  IV_Cruelty –CrueltyAppr-Internalisat-HM-B.R.  39.  IV_Cruelty –CrueltyAppr-Internalisat-AN-B.R.  40.  IV_Cruelty –CrueltyAppr-Internalisat-SH-B.R. | 21.IV_Injustice–Internalisation-B.R.  22.IV_Injustice–Humiliation-B.R.  23.IV_Injustice–Anger-B.R.  24.IV_Injustice–Shame-B.R.  25.IV_Cruelty–Internalisation-B.R.  26.IV_Cruelty–Humiliation-B.R.  27.IV_Cruelty–Anger-B.R.  28.IV_ Cruelty–Shame-B.R.  41.  IV_ Injustice –Internalisation-Humiliation-B.R.  42.  IV_ Injustice –Internalisation-Anger-B.R.  43.  IV_ Injustice –Internalisation-Shame-B.R.  44.  IV_Cruelty–Internalisation-Humiliation-B.R.  45.  IV_Cruelty–Internalisation-Anger-B.R.  46.  IV_Cruelty–Internalisation-Shame-B.R. |  |
| --- | --- | --- | --- |
